# Taking care to the patients: a qualitative evaluation of a community-based ART care program in northern Namibia

**DOI:** 10.1186/s12913-022-07928-0

**Published:** 2022-04-14

**Authors:** Leila Katirayi, Naemi Shoopala, Kiren Mitruka, Assegid Mengistu, Godfrey Woelk, Andrew L. Baughman, Gram Mutandi, Steven Y. Hong, Ndapewa Hamunime

**Affiliations:** 1 Research Department, Elizabeth Glaser Pediatric AIDS Foundation, Washington, D.C USA; 2 Ministry of Health and Social Services, Windhoek, Namibia; 3 Centers for Disease Control and Prevention, Atlanta, GA USA; 4 Centers for Disease Control and Prevention, Windhoek, Namibia

**Keywords:** HIV, Community-based ART, Namibia, Transportation, Community ownership, Main health facility

## Abstract

**Background:**

Namibia is a large sparsely populated country with a high prevalence of HIV. People living with HIV who reside in remote areas often travel long distances through tough desert terrain to access HIV care and treatment. To address this barrier, community-based antiretroviral therapy (C-BART) sites were established in Okongo (2007–2008) and Eenhana districts (2016) of northern Namibia with the goal of bringing HIV and other health services closer patients’ homes. We conducted a qualitative evaluation of the acceptability and challenges of C-BART to guide program improvement.

**Methods:**

For this qualitative descriptive study, research assistants collected data (August-December 2017) through in-depth interviews with 40 patients, seven health extension workers, and 11 policy/program managers, and through four focus group discussions with healthcare workers. Interviews were audio-recorded, translated, and coded using MAXQDA v.12. Data were analyzed using thematic analysis.

**Results:**

The evaluation identified five themes: community ownership, acceptance of the C-BART sites, benefits of the C-BART program for the PLHIV community and their social networks, benefits of the C-BART program to the main health facility, and challenges with the C-BART program. The C-BART program was reported as life-changing by many patients who had previously struggled to afford four-wheel drive vehicles to access care. Patients and healthcare workers perceived that the community as a whole benefited from the C-BART sites not only due to the financial pressure lifted from friends and family members previously asked to help cover expensive transportation, but also due to the perception of diminished stigmatization of people living with HIV and improved health. The C-BART sites became a source of community and social support for those accessing the sites. Healthcare workers reported greater job satisfaction and decongestion of health facilities. The challenges that they reported included delays in authorization of vehicles for transportation to C-BART sites and lack of incentives to provide services in the community.

**Conclusion:**

The C-BART program can serve as a model of care to expand access to HIV care and treatment and other health services to populations in remote settings, including rural and difficult-to-reach regions. The needs of healthcare workers should also be considered for the optimal delivery of such a model.

**Supplementary Information:**

The online version contains supplementary material available at 10.1186/s12913-022-07928-0.

## Background

Namibia, a country in sub-Saharan Africa, has made tremendous progress toward controlling the HIV epidemic with a 22% decline in AIDS-related mortality and a 36% decline in incidence over the last decade [1]. Globally, Namibia has one of the highest HIV prevalence rates (11.5%), with an estimated 210,000 adults aged ≥ 15 years living with HIV [2]. As of December 2020, Namibia was estimated to be at 90–98-91, as defined by the UNAIDS 95–95-95 treatment cascade, making them one of the first high-burden countries to approach epidemic control [3].

With a population density of 2.8 people per square kilometer, Namibia is among the most sparsely populated countries in the world. Half of its population of 2.3 million people reside in rural areas. People living with HIV (PLHIV) residing in remote areas often travel long distances through tough terrain to reach healthcare facilities, facing a substantial barrier in accessing HIV care and antiretroviral therapy (ART). Nationally, approximately 56.2% of households are estimated to live more than five kilometers from a health facility, with some 5% of households even requiring travel greater than 40 km to reach the facilities [4]. Some areas can only be reached by 4-wheel drive vehicles, which can cost between $20-$30 USD per trip. Only 6.4% of households own a motor vehicle and 30.5% have no access to a motor vehicle [4]. To address this distance challenge in Ohangwena, a rural region in northern Namibia with the second-highest regional HIV prevalence (17.9%) [5], a community-based ART (C-BART) program was implemented in the districts of Okongo in 2007 and in Eenhana in 2016. The goal of this program was to make ART more accessible by bringing HIV services closer to patients’ homes. Previous research has highlighted the success of C-BART programs in other low-income countries, including improved health outcomes and reduced patient burden on health facilities [6, 7].

The nurses serving the health facility in the Okongo District spearheaded the implementation of C-BART in close collaboration with community leaders and patients. In this area, where the main economic activity was subsistence agriculture, local community leaders provided the land and HIV-positive community members built and maintained the C-BART sites. A team from the facility comprised of one or two healthcare workers (HCWs), usually registered nurses, and a community counselor traveled to a C-BART site once every two months to provide services. A data clerk, tuberculosis field officer, and/or pharmacist assistant would also join the team, if needed. Core services provided by the team were ART prescriptions refills, HIV testing and counselling, clinical evaluations, blood specimen collections for viral load (VL) and CD4 analysis, and adherence counselling. Patients who missed appointments were followed up in their communities by health extension workers (HEWs), who were community-based health assistants employed by the Ministry of Health and Social Services (MoHSS). In Okongo District, patients were eligible to be down-referred from the facility to receive HIV care at a C-BART site if they lived close to a site and were willing to receive care there. In the Eenhana District, with similar economic activity to the Okongo District, patients were also required to be on ART for at least six months and have a suppressed VL to receive care at a C-BART site.

In 2017, the MoHSS, in collaboration with the Centers for Disease Control and Prevention (CDC) and the Elizabeth Glaser Pediatric AIDS Foundation (EGPAF), conducted an evaluation of the C-BART program in the Okongo and Eenhana districts to guide program improvement and inform scale-up. As of December 2017, 16 sites had been established in the Okongo District and 18 sites in the Eenhana District. This paper describes the qualitative results of the evaluation to provide an in-depth understanding of the acceptability and impact of the C-BART program on patients and healthcare facility staff.

## Methods

### Study design and population

We undertook a qualitative descriptive study using in-depth interviews (IDIs) and focus group discussions (FGDs) from August through December 2017. Participants included patients accessing C-BART sites, HCWs providing services at the C-BART sites, HEWs from the C-BART communities, and policy makers and program managers involved in the design and implementation of the C-BART program. See Table 1 for inclusion criteria.

**Table 1:** Inclusion criteria of participants in the qualitative evaluation of C-BART program

Population	Inclusion criteria
Patients	C-BART patients 18 years of age and older utilizing the C-BART sites on the days of data collection
HCWs	HCWs providing C-BART services for at least one month
HEWs	HEWs providing support for C-BART services for at least one month
Policy makers or program managers	Policy makers involved in the inception and program managers involved in implementation and support of the C-BART program

### Data collection

Based on review of the literature, we developed semi-structured individual interview and FGD tools and pretested them at non-evaluation sites with participants similar in characteristics to those in the evaluation, prior to official data collection. To ensure fidelity to the IDI process and data quality, the evaluation coordinator reviewed recordings of the first set of IDIs and FGDs from each research assistant (RA), providing feedback and guidance as necessary. The evaluation coordinator also reviewed a subset of the IDIs, accounting for 20% of the total, for quality assurance. IDIs with patients focused on their satisfaction and challenges with receiving services at the C-BART sites, perception of stigma, and challenges with adherence. FGDs with HCWs explored their views about the C-BART program, including its effect on patients’ ART adherence, and the successes and challenges of providing service at the C-BART sites. IDIs with HEWs focused on the community perspective and acceptance of the C-BART program. The IDIs with the program managers/policy makers reviewed the conception and implementation of the C-BART program to understand the processes involved. All discussions explored how the C-BART program could be improved.

All IDIs and FGDs were conducted by trained, Oshiwambo-speaking (the language spoke in northern Namibia) RAs. The training was conducted over a two-week period, which included extensive time roleplaying IDIs and FGDs. The RAs traveled to C-BART sites with the HCWs during scheduled visits to conduct IDIs with patients. Upon arrival, the HEW introduced the research team members and the purpose of the evaluation to patients waiting to be seen by the health facility team. IDIs with patients and HEWs were conducted in the local language of Oshiwambo. The FGDs with the HCWs and the IDIs with the policy makers/program managers were conducted in English at the participants’ preference. All IDIs and FGDs were audio-recorded. The encrypted and password-protected audio recordings were downloaded to and stored electronically on the study server. Identifiable data were not collected during the interviews; unique IDs were created for the participants and assigned at the time of the interviews.

### Site selection

The team of HCWs visited C-BART sites approximately every three months. Based on these scheduled visits, sites were selected for data collection in the two districts. A total of eight sites were visited, four in Eenhana and four in Okongo.

### Sampling and sample size

We employed a mix of convenience and purposive sampling. Patients were allowed to volunteer (convenience sampling) or were purposively selected if they were the first or last patients to receive services (to avoid losing their spot in the queue). RAs were instructed to ensure that at least one male participant per site was interviewed to ensure that the experiences of men were captured.

HCWs were selected using convenience sampling. To identify HCWs to participate in FGDs, the research evaluation coordinator requested volunteers, including nurses, data clerks, pharmacists, and counselors, at a monthly C-BART site meeting. Days and times for the FGDs were determined based on HCW preference. FGDs were conducted at the main health facility and consisted of 6–10 participants.

The district primary healthcare supervisor invited HEWs, identified by C-BART sites as having provided services for the longest period of time, to join the evaluation voluntarily. HEWs were interviewed at the main sites when they came to submit their reports at the end of the month.

The evaluation coordinator invited policy makers and program managers who were healthcare professionals and administrators at district, regional, or national policy levels involved in the C-BART program’s conception, planning, technical support, or program monitoring to participate in the evaluation. The participant groups’ sample sizes are presented in Table 2.

**Table 2:** Sample size of participant groups in the qualitative evaluation of C-BART program

Participants	Number of IDIs and FGDs
Patients	40 IDIs (20 in Okongo/20 in Eenhana)
HCWs	4 FGDs – with a total of 26 participants (2 in Okongo/2 in Eenhana)
HEWs	8 IDIs (6 in Okongo/2 in Eenhana)
Policy makers and program managers	11 IDIs - 3 at the national level - 4 at the regional level - 4 at the district level

The sample size of each participant group was based on achieving “saturation”, or when no new or additional information is expected from further interviews or discussions. Previous literature has indicated that saturation is reached at 10–12 IDIs and at 3–6 FGDs per homogenous group [8, 9]. In our study we considered patients, HCWs, and HEWs to be separate “homogenous” groups. A higher number of patient IDIs were planned because patient acceptability of the C-BART program was a primary objective and their group was expected to have greater variation among participants in age, gender, health status, and duration of HIV care. HCWs from multiple facilities and different cadres were included in the FGDs. Given that HEWs with longer experience of providing services at the C-BART sites were purposively selected, 6 of the 8 HEW IDIs were conducted in the Okongo District, since the C-BART program was implemented here earlier than in the Eenhana District. For the program/policy makers IDIs, 3–4 individuals from varying staff levels were interviewed to capture varied experiences and perspectives in designing and implementing the program.

### Data management and analysis

Transcripts were analyzed using thematic analysis. The audio recordings were translated to English (if conducted in Oshiwambo), and transcribed into Microsoft Word by the RA that conducted the interview. To assure quality translation, the evaluation coordinator reviewed 20% of the interview translations and transcriptions. Transcripts were reviewed by the lead qualitative investigator to generate a code list, a list of labels and phrases identified through reoccurring text. The codes were then compiled under a higher-level of categorization called themes. The code list was revised several times as new codes were identified during the coding process. Transcripts were coded by two RAs in the qualitative software program MAXQDA v.12. Code reports were generated for each participant group and code. From the code reports, data reduction and summary tables were created by two RAs under the supervision of the evaluation team to help identify recurrent patterns and themes for patient satisfaction, experiences of HCWs providing care, and recommendations for improvement. Text excerpts were identified that were representative of the themes identified.

### Ethical considerations

All research staff were trained and certified in the protection of human subjects and signed a confidentiality agreement prior to interacting with participants. Written informed consent, which included consent to be audio-recorded, was obtained from all evaluation participants prior to data collection. The evaluation received ethical approval from the MoHSS Research Ethics Committee in Namibia and Advarra Institutional Review Board in the US, a service contracted by EGPAF. The protocol was also reviewed in accordance with the CDC’s human research protection procedures, and was determined to be research, however the CDC investigators did not interact with human subjects or have access to identifiable data or specimens for research purposes.

## Results

### Demographic characteristics of participants

Table 3 presents the demographic characteristics of the patients, HCWs, and HEWs. Over half of the patients interviewed were 45 years of age and older, married, female, and had a primary level or less of education. Compared to the general patient population of the C-BART program, the evaluation had less patients under the age of 35 (5% versus 31%), a higher proportion of patients aged over 45 years (55% versus 27%), and more patients who were married (58% versus 40%). The gender distribution among patients in the evaluation was similar to the overall C-BART population [10].

**Table 3:** Demographic characteristics of participants in the qualitative evaluation of C-BART program

Characteristic	Patients	HCWs	HEWs
	(*n* = 40)	(*n* = 26)	(*n* = 7)^ab^
**Age**			
25–34 years	2 (5%)	15 (57.7%)	5 (71.4%)
35–44 years	16 (40%)	9 (34.6%)	1 (14.3%)
45 + years	22 (55%)	2 (7.7%)	1 (14.3%)
**Sex**			
Male	15 (37.5%)	8 (30.8%)	1 (14.3%)
Female	25 (62.5%)	18 (69.2%)	6 (85.7%)
**Education**			
No school	11 (27.5%)	0	0
Primary	25 (62.5%)	0	0
Secondary	4 (10%)	17 (65.4%)	7 (100%)
Tertiary	0	9 (34.6%)	0
**Marital status**			
Married/lives with partner	23 (57.5%)	^a^	^a^
Never married	9 (22.5%)	^a^	^a^
Separated/divorced	2 (5%)	^a^	^a^
Widowed	6 (15%)	^a^	^a^

^a^Question not asked of this group

^ab^Demographic data missing for one HEW

The majority of HCWs and HEWs were less than 35 years old. The HCWs had at least a secondary level of education, while all of the HEWS had exactly a secondary level of education. Data on the 11 policy makers and program managers was only collected on sex, with similar numbers of male (*n* = 6) and female (*n* = 5) participants.

The majority of HCWs were health assistants (*n* = 9), followed by nurses (*n* = 5); the remaining 12 consisted of the following roles: pharmacy assistant, laboratory technician, community health worker, data clerk, and tuberculosis field promoter.

The analysis revealed five themes in the data: community ownership, acceptance of the C-BART sites, benefits of the C-BART program for the PLHIV community and their social network, benefits of the C-BART program to the main health facility, and challenges with the C-BART program.

### Community ownership of the C-BART sites

One of the most important aspects for the establishment of the C-BART sites was the level of community involvement and ownership. Community leaders provided land for the erection of C-BART sites free of charge and encouraged their community members to utilize the sites.

*Leaders are the first individuals who agreed to have ART sites in the community and they provided land for these ART sites… they care for their people, they tried hard to have sites because health centers are very far…* (Patient 08, 43 year-old male, Eenhana)

Community members were responsible for building many of the sites from traditional materials, maintaining the cleanliness of the sites, and, in some instances, guarding them. The existence of the C-BART sites was a great source of pride for the communities.

*I really like the idea of a community site because now we feel proud, even when people from neighboring villages pass by here they can tell that our village is developing and that is a good feeling. Even us when we are checking we can see that the government is really working, they recognized us.* (Patient 52, 41 year-old male, Okongo)

### Acceptance of the C-BART sites

Patients initially struggled with the arrival of the C-BART sites, with fears of being seen accessing services at the sites and the potential for unintended disclosure of their HIV status. Attitudes towards the C-BART shifted, however, and the PLHIV community members became very welcoming of the C-BART sites and appreciative of the ability to access ART without the need for expensive travel costs and long travel days.

*there were those that did not want to come to the outreach at the beginning because they did not want to be seen. But as time goes they even realized that others are getting better and even those who were sick are fine now.* (Regional policy maker/program manager)

As patients realized the benefit of being able to access ART within their community, utilizing C-BART sites became viewed as a ‘privilege’ and patients were eager to remain adherent to ART and maintain a low VL to avoid being up-referred back to the district health facility.

*The program is normally for people that are on a normal viral load… If the viral load gets higher, she or he will be sent to the main facility again. Because they know that if they have high viral load they will be transferred to Eenhana, they take their medications well, well.* (HCW 06, FGD 01, Eenhana)

HCWs generally felt that the improved viral suppression rates and health conditions among C-BART patients were due to improved access to services with the arrival of the C-BART sites.

*Some patients, sometimes when they come to collect their medicine they used to be defaulters but the moment they were transferred to C-BART sites they are always present.* (HCW 04, FGD 02, Okongo)

Patients reported feeling that they were more likely to discuss their health concerns with HCWs at the C-BART sites, since they no longer felt rushed to begin their journey back home.

*[When accessing the main health facility at Okongo] my health was somehow jeopardized also because even if I did not feel well, I wouldn’t mention it because I just wanted to pick up my ART and go back and that is all brought by the fear of going back late but here [C-BART site] I am free and open because even if it gets late I will still air my complaints as there is nothing to fear.* (Patient 54, 47 year-old male, Okongo)

Another aspect of the C-BART program which may have influenced ART adherence and retention was the inclusion of the HEWs in 2015 in both the Okongo and Eenhana districts. The HEWs supported community members by encouraging them to attend the C-BART sites, reminding them of appointment dates, providing education to those struggling to take their medications, and providing a link between the C-BART teams and the communities.

*As a health extension worker, I am responsible to provide patients with information on how to take ART as prescribed also, to make them understand not to miss taking their ART. I normally walk house to house to see if they have ART and if they take them seriously and at prescribed time and also to encourage kids that also on ART to adhere to ART.* (HEW 01, Eenhana)

### Benefits of C-BART sites among PLHIV and their community

Previously, patients faced substantial financial pressure to secure money to travel to the health facility. To cover transport costs, participants discussed selling their livestock and borrowing money from friends and family. Patients and HCWs perceived that the community as a whole benefited from the C-BART sites due to the financial pressure being lifted from friends and family members previously asked to help cover expensive transportation.

I was spending amounts of money when I was going to Eenhana for ART, sometimes I had no money or could not find money then I had to borrow money for transport costs to the hospital. (Patient 08, 43 year-old female, Eenhana)

*I ended up having to sell my goats just to get transport money while people are dying of hunger at home.* (Patient 51, 58 year-old male, Okongo)

An unanticipated benefit of the C-BART sites was the generalized sense of a decrease in stigma within the community and an increase in openness about HIV testing and status among PLHIV.

*You can also see that stigma and discrimination is narrowing. People are free when they are coming. Other people are also coming to sell their kapana (grilled meat). The reason they are allowed to come there is because these people have peace of mind. No one has anything against these people. You can see that the level of understanding has gone up.* (HCW 06, FGD 01, Eenhana)

Meeting at the C-BART site also provided an opportunity for PLHIV to talk openly among each other.

Patients were supportive of each other, creating an environment of trust and encouragement.

*They support each other at the point, if there is someone who is not doing well on treatment they can even go and visit them to encourage them, they are even used their own example like “look at me, I started treatment when, now I am doing well, my viral load is suppressed” so patients… they are taking ownership of the service.* (HCW 01, FGD 02, Okongo)

Accessing medicine at the C-BART also increased patients’ self-confidence and motivation to live with an HIV-positive status.

*It also now brought self-motivation yeah, people that come receive their ART at the C-BART sites they are very confident and they are not shy anymore. Everywhere they go they testify that they are fine now they carry their pills with them without fearing to be seen as some of them are usually shy.* (HCW 07, FGD 01, Okongo)

Patients reported that the C-BART sites generally improved their quality of life by giving them more time to work, care for children, tend livestock, and address other responsibilities.

It’s very easy because coming to this site won’t affect my regular household work, which is good. (Patient, 05, 58 year-old female, Eenhana)

### Benefits of the C-BART program to the main health facility

HCWs observed decongestion in the district health facilities as patients were down-referred to the C-BART sites. Previously, community members would save up and share the costs of the 4-wheel transport to reach the health facility, which meant that large groups would arrive together and overwhelm HCWs.

*When the C-BART sites weren’t there, all those patients could have been coming here at the main site and the patient load could be extremely high which could put me on pressure but now since they are divided at different sites I am satisfied because I am never under pressure anymore.* (HCW 07, FGD 01, Okongo)

HCWs discussed feelings of job satisfaction knowing that their service at the C-BART sites determined whether patients received life-saving ART.

*whenever we get there [C-BART site] they have already gathered and some they would have even have traveled from far or put their health passports a day before hoping and waiting so when you get there… it is good, it gives you job satisfaction that they have faith in you and we in them.* (HCW 04, FGD 01, Okongo)

### Challenges with the C-BART program

All participant groups discussed the need for C-BART site infrastructure enhancements to improve privacy for discussions and physical exams and asked for upgrades from stick to pre-fabricated structures, some of which had already been implemented by PEPFAR and Namibia MoHSS.

I like the way they give provide us with good information, but commonly doctors and nurses cannot witness some of our illness as we just get our service in that tree, there are no private rooms here. (Patient, 02, 35 year-old female, Eenhana)

All participant groups discussed the challenge of enduring the rain and strong sun under the stick C-BART structures and absence of proper waiting areas.

*During the rainy season, we will be forced to be in a car and the patients will be there and the rain will be all over them… Even the papers and books we are using will be spoiled by the rain.* (HCW 05, FGD 01, Eenhana)

Patients complained about the HCWs arriving late or packing up early before all patients had arrived and received their services, which resulted in having to travel to the district health facility to receive their ART.

*I noticed that other patients from far places can go without their ART when they come late… some healthcare workers can see that patients are coming but they just pack and go without providing services to them as a result those clients will again walk to Eenhana for their ART.* (Patient 01, 48 year-old male, Eenhana)

HCWs stated that there was no designated vehicle for the C-BART visits. Therefore, on some days it took time to organize a vehicle and a driver, resulting in delays in arriving to the sites to provide services.

*sometimes we go very late… sometimes there is no transport that is really [dedicated] for CDC, the car for outreach. When you go there sometimes, there is no driver and you say “let me find a driver” and there is no driver available for you. By the time you go there, it’s late and then the patients are complaining. “No, you took so long”. It’s true but we have nothing to do. If we could have our own transport, just for ART, it could be better. People [HCWs] could go early in the morning.* (HCW 05, FGD 01, Eenhana)

The vehicles used to transport HCWs to the C-BART sites were also insufficient. HCWs advocated strongly for designated C-BART vehicles, outfitted with a cooler box for sample storage and a water tank, so that HCWs are able to wash their hands after each patient.

*There is this thing of taking the blood, and there is something that says after the blood is withdrawn, how many hours it needs to be reported [deposited at lab]? A lot of our outreach sites are very far. When we get here [main facility], those hours have already passed.* (HCW 02, FGD 01, Eenhana)

HCWs also discussed that the current working conditions at C-BART sites were not favorable, nor in line with government standards. HCWs discussed that previously they received lunch packs and a bush allowance (an amount that is given to government employees that work in remote areas), but this benefit had stopped.

*Suspending the lunch packs is a mistake that has been done. We should be able to make these people [HCWs] happy when they are going there [C-BART sites] because they are going out of their comfort, they are going out of where they are supposed to work... Although, we go in the community, we even go further than those who are paid bush allowance. What we are saying is; they should take care… they should not ignore them [HCWs] when they are improving the services of the patients.* (HCW 06, FGD 01, Eenhana)

HCWs also felt they should be given the choice of whether they wanted to work at C-BART sites and should be able to opt out of providing services at the C-BART sites, as some HCWs did not want to be working at them.

*but I just have to go there [C-BART site] because I don’t have a choice, but it is not something that I want to do, it takes me out of my comfort zone, and it’s sunny when you come back your clothes are dirty, its windy even.* (HCW 05, FGD 02, Eenhana) E-H-02

## Discussion

We found that the C-BART program was well-accepted among all participants groups and patients reported an improvement in their quality of life, as the burden of traveling to healthcare facilities to access ART was lifted. In fact, HCWs and HEWs noted an observed improvement in both ART adherence and retention, suggesting that the C-BART program achieved its intended health outcomes.

Geographic and transportation-related barriers have been documented to be associated with poor outcomes across the continuum of HIV care [11]. HCWs, HEWs, and patients stated that the transportation fees required to reach the district health facilities was unaffordable for most patients and served as the main cause of loss to follow-up and poor adherence. Previous models of care have attempted to address the transportation challenges, but not all models have been successful in retaining patients in care. For example, while community adherence groups have been successful at lowering patient transport costs and building peer support, internal group dynamics of the local groups have been reported to result in patients leaving the community adherence groups and returning to conventional care [12]. Home-based care models have been shown to improve health outcomes and reduce attrition [13], but the desert and vast terrain of northern Namibia would have presented transportation challenges, requiring substantially more resources. Another model from South Africa provided patients with multi-month dispensing with six months of ART to reduce patient travel time to clinics. Results found that participants reported greater motivation and ART adherence, ‘peace of mind’, and a better sense of control over their treatment [14]. These finding were similar to patient reports from the C-BART program and highlight the importance of better integration of HIV care in patients’ lives. An important element of C-BART program that was not intentionally designed but may have been a key factor in the acceptability of C-BART sites, was the emergence of a social support structure. The coming together of the HIV community at C-BART sites fostered the development of a support network for patients to discuss their concerns, exchange solutions, and foster empathy with each other. A qualitative systematic review found that social support was a critical aspect in maintaining ART adherence. Social support helped PLHIV gain self-confidence and a sense of belonging, reducing the effects of stigma [15]. A study comparing clinic versus community-based ART clubs in Johannesburg, South Africa found that in the ART clubs, patients felt a new ‘openness’ or ‘freedom’ to discuss HIV-related issues and the groups led to peer support and friendships that empowered individuals to accept their HIV status and take their ARTs [16]. Another study that also explored community-based ART groups, found that social and peer support significantly improved individuals’ ability to remain motivated to continue their ARTs and visit each other when ill [17]. Overall, community-based interventions have been shown to increase HIV awareness and risk-reduction [18].

Patients reported feeling more comfortable receiving services at the C-BART sites compared to the main health facility, which could explain their retention in care at these sites. Preliminary findings from the quantitative evaluation of these C-BART sites showed 24- and 60-month retention among adults to be approximately 96% and 91%, respectively [10]. These rates were similar to the MoHSS retention estimates for these Districts, and were significantly higher than the national retention estimates of 83% at 24 months, and 70% at 60 months [19]. A similar view has been expressed by patients about community-based ART clubs, which were felt to provide more attentive care compared with that of the facility [20]. Furthermore, HEWs likely enhanced C-BART services as they followed up with patients who missed appointments, provided support to patients, and facilitated communication with district healthcare facilities regarding barriers to continuation of treatment. Previous research has found that the psychosocial support offered through community health workers is critical to reducing patient attrition [7].

The concern for stigma related to disclosure of HIV status has been a reason why HIV-positive patients have preferred facility-based care over community-based care [21, 22]. While stigma was an initial concern at C-BART sites, a change of attitude was observed over time after implementation. Participants said that prior to the arrival of C-BART sites, HIV had been viewed as a death sentence in the community, a sentiment that is well-documented in the literature [23–25]. Visibly improved survival by people living with HIV/AIDS seemed to reduce stigma. Stigma reduction has been documented to improve the quality of life and patient outcomes. A study in South Africa found stigma reduction for PLHIV resulted in a shift in self-judgement that led to behavior change, including fewer symptoms, improved treatment adherence, and improved relationships and quality of life [26].

The C-BART program was highly compatible with the values, experiences, and needs of the PLHIV they served due to the high level of community engagement in the design and implementation of the sites. The involvement of community leaders demonstrated the value and validation of the C-BART program to the community members. The PLHIV community members were involved from the beginning with building the site structures and maintaining them. In fact, ownership in the development of HIV interventions has been documented to result in better HIV awareness, decreased stigma, improved health seeking behavior, and better quality of care [27]. A systematic review of community-based ART programs found that to be successful, community programs need to be driven by, owned by, and embedded in the communities [28].

There were some significant challenges with the C-BART program that prevented it from operating at its optimum level. The inconsistent availability of vehicles for the HCW teams was a challenge that resulted in delays in reaching C-BART sites or encouraged them to leave the sites early. Having reliable transport, with the water and appropriate sample storage could significantly improve the reliability of the HCWs’ visit times and provide more desirable (and safer) working conditions. All participants requested that C-BART sites be upgraded from stick to pre-fabricated structures, to allow for more privacy to conduct physical exams and to help shelter from inclement weather. It is also important to recognize that not all HCWs wished to support the C-BART sites. This perspective could change with the addition of incentives, such as allowances and packed lunches.

With the “test and treat” approach, there has been an increased demand on the health facilities. To meet this need, it will be necessary to identify methods to improve efficiency at the health facilities to cater to the high volume of patients while simultaneously improving patient health outcomes. One approach is to focus on decentralizing healthcare services, like the C-BART program has done, which not only reduces the burden on facilities, but also ensures patients in distant locations are able to access healthcare services. Non-adherent patients cost countries more by developing treatment resistance and requiring more expensive second- and third-line ART regimens. The long-term costs saved by implementing new strategies may outweigh the initial high costs of patients-centered services.

## Limitations

Patients included in this evaluation were restricted to those accessing care at the C-BART sites during the evaluation visits and were not representative of the overall C-BART population, limiting the generalizability of the findings. Patients who had temporarily received care at the C-BART sites, but had either dropped out of the program or been referred back to the district facilities were not captured and might have expressed different opinions. Furthermore, the inclusion of all participants was based on volunteerism, which could have introduced selection bias.

## Conclusion

Participants in this evaluation uniformly reported that the C-BART program was highly accepted and appreciated due to the benefits experienced by patients. Though it had some implementation challenges, the C-BART program provided patient-centered care and improving HIV outcomes. The C-BART program in Namibia can serve as model of care to expand access to HIV care and treatment and other health services to populations in remote settings, including rural and difficult-to-reach regions.

## Supplementary Information

**Additional file 1.** 

**Additional file 2.** 

## Data Availability

Data are available from the authors upon reasonable request and with the permission of the Namibia MoHSS.

## Abbreviations

ART: Antiretroviral therapy
C-BART: Community-based ART
CDC: Centers for Disease Control and Prevention
EGPAF: Elizabeth Glaser Pediatric AIDS Foundation
FGD: Focus group discussion
HCW: Healthcare worker
HEW: Health extension worker
IDI: In-depth interview
MoHSS: Ministry of Health and Social Services
PLHIV: People living with HIV
RA: Research assistant
VL: Viral load
