# Supplementary material for: Taking care to the patients: a qualitative evaluation of a community-based ART care program in northern Namibia
Source: BMC Health Serv Res. 2022 Apr 14;22:498. doi: 10.1186/s12913-022-07928-0 (PMC9009034; doi:10.1186/s12913-022-07928-0)
Supplement: Supplementary file 1 — Additional file 1. [file 12913_2022_7928_MOESM1_ESM.docx]

# Interview & FGD Guides

## Interview Guide for Patients

Participant number __ __ __ __

Interviewer name: ________________________________

Site number: __ __ (*pre-assigned number*)

Visit date: __ __ / __ __ / __ __ __ __ (*dd-mm-yyyy*) Start time: _____: ______

*INSTRUCTIONS:* *This* *interview should only be started once informed consent has been obtained from the participant. Use the probes as needed if the respondent looks uncertain or indicates that he/she is unsure what you mean. Interviewer instructions are in italic print—these are for your use and should not be read aloud.*

**Thank you again for agreeing to participate in our study.**

**Demographic Information**

A1. Gender

Male  (1)

Female  (2)

A2. Date of birth______________ (If not known, Age: __ __ (years) (age at last birthday))

A3. What is your marital status?

Married  (1)

Never married  (2)

Separate  (3)

Divorced  (4)

Widowed  (5)

Living with partner  (6)

A4. What is your level of education?

No school  (1)

Some primary  (2)

Completed primary  (3)

Some secondary  (4)

Completed secondary  (5)

Some tertiary  (6)

Completed tertiary  (7)

A5. How long have you been on ART? _______ (months) ________ (years)

A6. How many times have you accessed the community site for ART? _______________

A7. What other services do you access at this site?

Pick up ART  (1)

Treat opportunistic infections  (2)

General exam  (3)

Test CD4 count  (4)

Test viral load  (5)

Treat TB  (6)

Nutrition assessment/support  (7)

Family planning  (8)

Screen for STIs  (9)

Screen for cervical cancer  (10)

Treat HIV associated  (11)

complications

A8. How do you normally get to the site? (Select one method only)

Walk  (1)

Bicycle  (2)

Private car  (3)

Bus  (4)

Donkey cart  (5)

Other  (6) Specify: ___________________________________

A9. How long does it take you to reach this community site? ____ (minutes) ______ (hours)

A10. Has there been a time that you could not access ART at the community site and had to travel elsewhere for ART?

No  (0)

Yes  (1)

A11. Do you ever pick up ART for others?

No  (0)

Yes  (1)

**Accessibility of the Community ART Sites**

1. How easy for you is it to access services at this community site? (*Probe to understand if the location is easily accessible, if they receive enough notice that the health care team is visiting, if the hours that the community site is open are okay, etc.*)
2. Once you arrive at the community site, how easy it is for to utilize services at this community site? (*Probe – queuing, time to receive services, always receive the services they came for, issues with stock-outs, etc.*)
3. What are the services that you utilize at this site? (*Probe to learn about ALL services that they receive from this site including drugs, check-ups, counseling, support, etc.*)

**Barriers/Facilitators to Utilizing the Community ART Sites**

1. What are some of the challenges you face utilizing services at this community site? (*Examples could include not having enough time to be seen by the doctor, lack of privacy, not receiving all the services needed, challenges with schedule of visits, needing more services that are offered at the community sites, etc.*)
2. How have you solved any of these challenges?
3. Are there any financial costs for to use services at this site? (*Probe – costs of transportation, bringing medical supplies, medication costs, taking time off work to come to the site, etc.*)
4. What are some of the benefits of utilizing services at this community site? (*Probe – easy to access, able to get multiple services done in one place, etc.*)

**HCWs Providing Services**

1. What do you think about the health care workers providing services at the community site? (*Probe: Friendliness? Trust their advice? Feel that you receive the information and support you need?*)

**Effect of the Community ART Sites on Adherence**

1. How have the community sites affected your ability to adhere to ART? (*Probe: If the community sites were not here, do you think it would affect your ability to adhere to ART?*)
2. *Question for those who initiated ART before 2007 –* How has the arrival of the community sites affected your ability to adhere to ART? (*Probe to understand if adherence has strengthened with the arrival of the community sites or if it has stayed the same or weakened.*)

**Personal Perspectives towards the Community ART Sites**

1. What are some of the things that you like most about this community site? (*Probe – convenience of location, ability to receive ART nearby, able to get services needed, etc.*)
2. What are some of things that you don’t like about this community site? (*Probe – not enough services provided, no privacy, not enough time to receive services, location is inconvenient, no waiting space, etc.*)
3. If you could improve the community sites, what changes would you make? (*Probe – more services offered, more frequent visits, longer hours, more health care providers, more privacy, more frequent notice of upcoming visits, etc.*)

**Community Perspectives toward the Community ART Sites**

1. What is said in the community about these community sites? (*Probe – messages from leaders, what elders say, what community members say, what the youth say, etc.*)
2. Please tell me about any stigma associated with using the community site? (*Probe – being seen utilizing site, stigma in the community towards the site, etc.*)
3. Has stigma affected your use of the community site? If yes, please tell me how it has affected you.

**Recommendations to Improve the Community ART Program**

1. What recommendations do you have to improve the community program? (*This is very open, can be about the location of site, physical space in which services are provided, what services should be provided, attitudes toward health care workers, etc.*)

**This is the end of our interview. Thank you very much for taking the time to participate in this interview.**

End time: ____: _____

## Interview Guide for Key Informant Interviews - Policy Makers and Program Managers

Participant number __ __ __ __ Site number: __ __ (*pre-assigned number*)

Interviewer name: ________________________________

Visit date: __ __ / __ __ / __ __ __ __ (*dd-mm-yyyy*) Start time: _____: ______

*INSTRUCTIONS:* *This* *interview should only be started once informed consent has been obtained from the participant. Use the probes as needed if the respondent looks uncertain or indicates that he/she is unsure what you mean. Interviewer instructions are in italic print—these are for your use and should not be read aloud.*

**Thank you again for agreeing to participate in our study.**

**Demographic Information**

A1. Type of key informant

Policy maker  (1)

Program manager  (2)

A2. Gender

Male  (1)

Female  (2)

A3. Age:

>30

31-40

41-50

50+

A4. Involved with which district:

Okongo  (1)

Eenhana  (2)
Both  (3)

A5. Place of employment (specify district, regional levels):

________________________________________________________________________________

A6. Current title:

________________________________________________________________________________

A7. How many years have you been a policy maker/program manager?

_______________ (years)

A8. How were you involved in the creation of the C-BART program? *(Select all that apply)*

Conception of idea  (1)

Design of C-BART  (2)
Planning of C-BART  (3)

Implementation  (4)

Reporting data  (5)
Other (describe): ________________________________________

**Conception of C-BART Sites: POLICY MAKERS**

1. Can you tell me how the idea of having community-based ART sites was conceived?
2. Who was involved in this process?
3. Was anyone else consulted? If so, who?
4. Who (which groups/stakeholders) initially supported the idea, and who (which groups) did not support the idea?
5. Why did these groups oppose the idea? What were their concerns?
6. For the groups that were supportive of the idea, what were their reasons for supporting the idea?
7. In the end, how did you gain support for the idea to at least be tested?
8. Who approved the idea to be implemented?
9. After getting support for the idea/concept, can you tell me how the idea was implemented?
10. At the national level, who was responsible to implement the program?

**Design and Planning of the C-BART Program: POLCY MAKERS/PROGRAM MANAGERS**

1. Tell me about the process of designing, planning and implementing the program. *For example, who was consulted in its design and implementation? Where did the resources come from for the program, including the staffing? Who designed the reporting/M&E system? Was there any training involved? Who was trained and who provided the training?*
2. What role did various interest groups and stakeholder play in the design and implementation of the program?
3. How were the local communities involved?
4. Please describe the current system of the C-BART sites. (*Probe: Who are the different groups (such as HCWs, community leaders, etc.) involved in the C-BART sites? How is communication managed? Who is in charge of the program?*)
5. What were the initial community responses when the program was first implemented?
6. How has the arrival of the C-BART sites changed attitudes towards HIV in the community? (*Probe to understand any effects of the C-BART sites – HIV discussed more openly, community more knowledgeable about HIV, development of support groups, development of additional services or program for people living with HIV*)
7. How has the community attitude towards the C-BART sites changed over time?
8. How did the healthcare workers initially feel about working in the C-BART sites? (*Probe: How has this attitude changed?*)
9. Please describe how the health extension workers (HEWs) work with the sites? (*Probe: How do the HEWs communicate with the HCW teams at the C-BART sites? What is the role of the HEW in supporting the C-BART sites?*)
10. How do you think the presence of the C-BART sites has affected patient’s adherence? (*Probe: What has been said by HCWs providing the services? What has been said by community leaders? What data has the participant seen to indicate how adherence may be affected?*)
11. What have been some the challenges experienced implementing the C-BART sites? (*Probe for the different types of challenges: financial, staffing, coordination, communication, logistics (like transportation), ownership, political, etc.. With this question, be sure to clarify who was experiencing the challenge (community, HCW team, main ART site, etc.) Please take notes on this.*)
12. How have these challenges been resolved? (*Probe – refer to your notes and ask about the solutions for each of the problems mentioned. If there was no solution, ask what possible solutions were considered and why they were not implemented.*)
13. Has the C-BART program affected the programs at the clinics and hospitals? If so, how have they been affected?
14. In your opinion, what can be done to strengthen the C-BART programs? (*Probe for the different areas: staffing, coordination, scheduling, communication, physical sites, organization (such as transportation), services offered, costs, etc.*)
15. If this program was being implemented in another district, what are the three most valuable pieces of advice you would give a new district implementing community-based ART?

**We have reached the end of our interview. Thank you for your time.**

**End time: ____: _____**

## Interview Guide for Health Extension Workers

Participant number __ __ __ __

Interviewer name: ________________________________

Site number: __ __ (*pre-assigned number*)

Visit date: __ __ / __ __ / __ __ __ __ (*dd-mm-yyyy*) Start time: _____: ______

*INSTRUCTIONS:* *This* *interview should only be started once informed consent has been obtained from the participant. Use the probes as needed if the respondent looks uncertain or indicates that he/she is unsure what you mean. Interviewer instructions are in italic print—these are for your use and should not be read aloud.*

**Thank you again for agreeing to participate in our study.**

**HEWs’ Roles Providing Services**

1. Please describe your role and specifically how you work with the community-based ART programs. (*Probe: How do they support the community sites, what services do they provide, track patients, refer patients to the sites, deliver medicine from the sites to the patients, etc.*)
2. Please describe your communication with the health care workers who service the community ART sites (*Probe: How do they communicate with the HCW teams? Is it via through the community leaders? How do they know when the health care workers are coming to the sites, etc.?*)

1. Please describe if you were trained or oriented in supporting the C-BART program? Would you require any other training to strengthen your role in supporting the C-BART program? If so, please describe what areas you would require training in.

**Patients Utilizing the Community ART Sites**

1. Who are the general patients accessing the community site? (*Probe to get a sense of if it is more men, women, pregnant ladies, adolescents, children, etc.*)
2. Do you think that there are any community members who are not adequately accessing the community sites? If yes, please describe who and what can be done to attract these others to utilize the community sites.
3. For those who have been working for more than two years as a health extension worker to support the community ART program, what changes have you seen with uptake of services from the community ART program since you started supporting it? (*Probe – increased client flow, increase in different types of services requested, more community acceptance of the program, etc.*)

**HEWs’ Perspectives/Attitudes towards the Community ART Sites**

1. What do you think are some of the main successes of the community ART sites?
2. What do you think are some of the main challenges with the community ART sites?
3. Do you think the types and amounts of services (i.e. provision of ART refills and education, etc.) currently provided at the community sites are sufficient? Why do you think so? Are there any other services that you think should be provided? If yes, please specify?

**Patients’ Perspectives/Attitudes towards the Community ART Sites**

1. In general, how do you think the patients feel about the community sites? What have they said about it?
2. What are of the complaints that you have heard from patients about utilizing the community sites?
3. What are some of the positive things that patients have said about the community sites?
4. How do you think the community sites have affected patients’ ability to adhere to ART? (*Probe: If the community sites were not here, do you think it would affect patient’s ability to adhere to ART?*)

1. *Question for HEWs who were residents in the community and familiar with the ART program before 2007 –* How has the arrival of the community sites affected patients’ ability to adhere to ART? (*Probe to understand if they think adherence has been strengthened with the arrival of the community sites or if it has stayed the same or weakened*)

**Community Perspective towards the Community ART Sites**

1. What is said in the community about accessing the community sites? (*Probe: What is said by community leaders, community elders, and the community members?*)
2. Please tell me about any issues with stigma associated with the community ART sites. (*Probe to get a sense if stigma is a problem or not, if stigma discourages people from accessing the sites, how have community members have responded to the stigma, how community leaders have responded to stigma, etc.*)

1. For those who have been resident in the community and familiar with the ART program before 2007, what changes have you seen in terms of the community’s attitude towards the community ART site?

**Challenges Providing Services in the Community ART Sites**

1. As a health extension worker in the community ART sites, what are some of the challenges that you experience supporting the community ART site? (*Probe – insufficient communication with community site, transportation challenges, not enough resources do your job, unclear supervision/guidance, not enough support from the community leadership, etc.*)
2. How can these challenges be addressed?

**Recommendations to Improve the Community ART Sites**

1. How can the community sites be strengthened? What changes would you recommend? (*Probe – services provided, schedule of site visit, number of HCWs servicing the community site, types of HCWs servicing the site, supplies, communication with community, etc.*)
2. In what way can the HEW program assist in further strengthening the C-BART program?
3. What changes have patients suggested?

**This is the end of our discussion. Thank you very much for your participation today.**

**End time: ____: _____**

# Focus Group Discussion Guide for Health Care Workers

| Date of the FGD | __ __ / __ __ / __ __ __ __ (dd-mm-yyyy) |
| --- | --- |
| Site number | __________________________________ |
| Research assistant number for leading FGD | __________________________________ |
| Research assistant number for note taking | __________________________________ |

FGD number: _________ Number of participants: __ __ Start time: __ __: __ __

**Introduction**

Introduce the moderator and notetaker. Explain that we are here to learn about the health care workers’ experiences of providing services in the community-based ART program and their perceptions of patients’ attitudes towards the community-based ART program. Explain that we want to learn from them so that we can better improve the HIV/AIDS program within and outside of Namibia.

Assign participant numbers to be referred to throughout the FGD (give each person a piece of paper with their number on it). Explain that this helps protect their privacy and makes it easier for the notetaker to capture what they said.

**Overview of Working in a Community ART Site**

1. Please describe your different roles in supporting the community ART sites. It would be great to hear from each of the different groups (nurses, doctors, psychosocial counselors/health assistants, lab technicians, pharmacist assistants and pharmacists). *Please make sure that not too much time is spent on this question, in order to have enough time for the rest of the tool.*

**Preparation/Training to Provide Services in a Community ART Site**

1. Did you receive any additional training to provide services in the community sites? If yes, please describe. *Be sure to hear from the different types of HCWs.*
2. For any of your roles, do you think any additional training is necessary to be able to provide services in the community sites? If yes, what kind of training would help? Let us hear from all different group members, nurses, doctors, psychosocial counselors/health assistants, lab technicians, pharmacist assistants and pharmacists.

**Patients Utilizing the Community ART Sites**

1. Who are the general patients accessing the community site? (*Probe to get a sense of if it is more men, women, pregnant ladies, adolescents, children, etc.*)
2. Do you think that there are any community members who are not adequately accessing the community sites? If yes, please describe who, why they may not be accessing the community sites, and what can be done to attract these others to utilize the community sites.
3. For those who have been working the in the community ART program for more than two years, what changes have you seen with uptake of services from the community ART program since you started supporting it? (*Probe – increased client flow, increase in different types of services requested, more community acceptance of the program, etc.*)

**HCWs’ Perspectives/Attitudes towards the Community ART Sites**

1. Do you think the services currently provided at the community sites are sufficient? Why do you think so? Are there any other services that you think should be provided? If yes, please specify? (*Probe – frequency of C-BART scheduling, range of services provided*)
2. What impact do you think the community ART program has on individual’s ability to adhere to ART?
3. Has providing services at the community sites had any impact on your own personal professional satisfaction as a service provider? If so, please explain.
4. What do you think are some of the main successes of the community ART sites?
5. What do you think are some of the main challenges with the community ART sites?

**Patients’ Perspectives/Attitudes towards the Community ART Sites**

1. What are some of the challenges that you think patients experience when utilizing the community sites?
2. What helps the patients to utilize the community sites?
3. What are the complaints that you have heard from patients about utilizing the community sites?
4. What are some of the things that patients have said they like about the community sites?

**Challenges Providing Services in the Community ART Sites**

1. As a health care provider working in the community ART sites, what are some of the challenges that you experience providing care in the community ART sites? *Be sure to hear from each group.* (*Probe – stock-outs, insufficient space to provide services, lack of privacy for patients, insufficient training/preparation to work in the communities, safety issues, schedule challenges, travel challenges, too much work, too rushed, etc.*)
2. How can these challenges be addressed?

**Recommendations to Improve the Community ART Sites**

1. How can the community ART sites be strengthened? What changes would you recommend? (*Probe – services provided, set-up of the site, schedule of site visit, number of HCWs servicing the community site, types of HCWs servicing the site, supplies, communication with community, etc.*)
2. What changes have patients suggested?
3. For other sites thinking about starting community-based ART services, what would you say are the most important things to consider in setting up a successful program?

**This is the end of our discussion. Thank you very much for your participation today!**

**End time: ____: _____**
