# Supplementary material for: Taking care to the patients: a qualitative evaluation of a community-based ART care program in northern Namibia
Source: BMC Health Serv Res. 2022 Apr 14;22:498. doi: 10.1186/s12913-022-07928-0 (PMC9009034; doi:10.1186/s12913-022-07928-0)

Appendix.

Image 1- Traditional C-BART structure


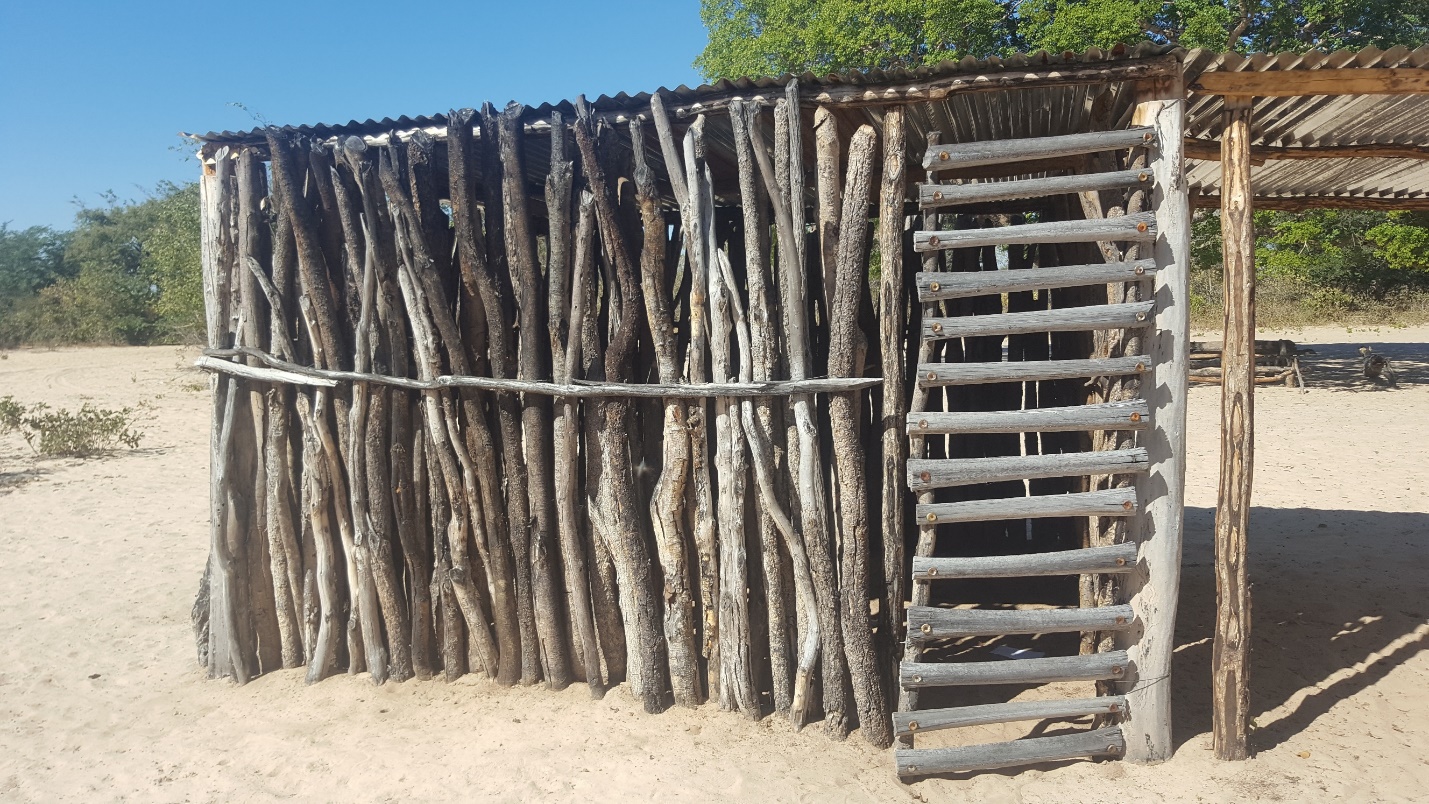


Image 2- New prefabricated structure


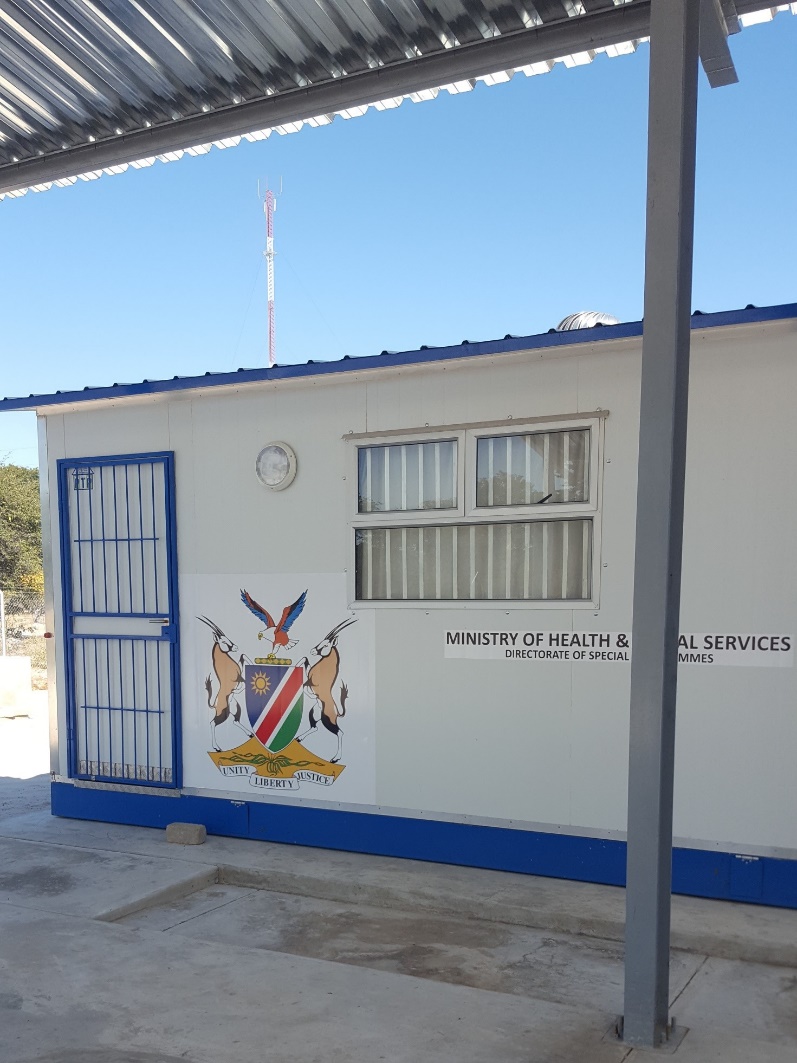


Image 3 - Unpaved road to Oshitishiwa C-BART site, Okongo District’


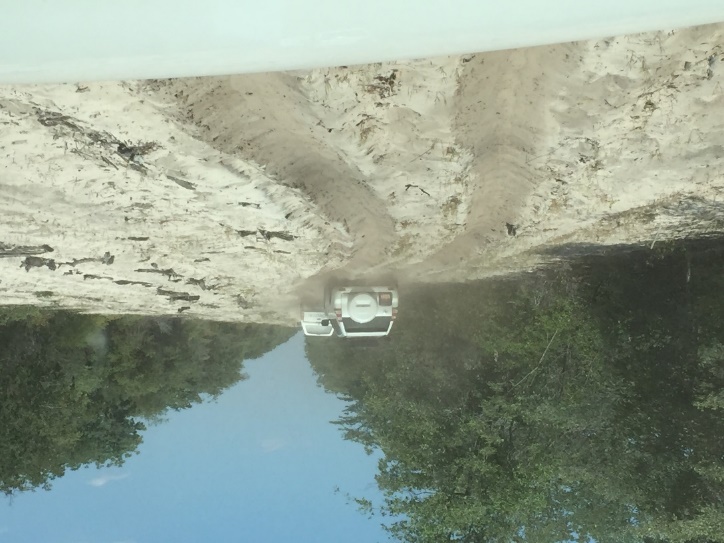

Supplement: Supplementary file 2 — Additional file 2. [file 12913_2022_7928_MOESM2_ESM.docx]
